# Pilot Study of Combined Tranexamic Acid Therapy in Breast Reduction – Intravenous and Local Infiltration

**DOI:** 10.1007/s00266-026-06024-6

**Published:** 2026-08-21

**Authors:** Margarida Silva, Pedro Machado, António Sousa Barros, António Costa-Ferreira

**Affiliations:** 1 Department of Surgery and Physiology, Faculty of Medicine, University of Porto, Porto, Portugal; 2 Department of Plastic, Reconstructive and Aesthetic Surgery, São João University Hospital, Porto, Portugal; 3 RISE-Health, Department of Surgery and Physiology, Faculty of Medicine, University of Porto, Porto, Portugal

**Keywords:** Breast reduction, Drain output, Suction drain, Edema, Postoperative complications, Reduction mammoplasty, Tranexamic acid

## Abstract

**Background:**

Tranexamic acid (TXA) reduces perioperative blood loss in several surgical fields; however, its role in breast reduction surgery remains unclear. This study aimed to evaluate the effect of combined TXA therapy (intravenous and local infiltration) on postoperative outcomes of patients who underwent modified Liacyr Ribeiro breast reduction.

**Methods:**

We conducted a controlled, non-randomized pilot study of patients who underwent bilateral reduction mammoplasty by the same surgeon. The intervention group received 1 g of intravenous TXA at anesthesia induction and local infiltration with a 1-mg/mL TXA vasoconstrictive solution containing epinephrine. The control group received local infiltration with an identical vasoconstrictive solution that did not contain TXA. Primary outcomes were total and daily drain output. Secondary outcomes included length of hospital stay, time to drain removal, and postoperative complications.

**Results:**

Fifty-seven patients were included (31 in the control group and 26 in the intervention group). TXA significantly reduced the total postoperative drain volume (*p*<0.001) by an estimated 79% and drain output at 24h (*p*<0.003). In the TXA group, 53.8 % of the patients had unquantifiable drainage versus 9.7% in the control group (*p*=0.002). No significant differences were observed in complications, including hemorrhagic events, infections, or wound healing; indeed, the incidence of complications was low in both groups.

**Conclusions:**

Combined TXA therapy significantly and clinically meaningfully reduced postoperative drain output. This reduction anticipates drain removal and may ultimately contribute to the broader adoption of drain-free breast reduction surgery.

**Level of Evidence II:**

This journal requires that authors assign a level of evidence to each article. For a full description of these Evidence-Based Medicine Ratings, please refer to Table of Contents or online Instructions to Authors www.springer.com/00266.

**Supplementary Information:**

The online version contains supplementary material available at https://doi.org/10.1007/s00266-026-06024-6.

## Introduction

Tranexamic acid (TXA) is a synthetic lysine analog and an affordable antifibrinolytic drug that inhibits the conversion of plasminogen to plasmin, thereby preventing fibrin clots dissolution and ultimately reducing bleeding [1]. It also exhibits anti-inflammatory effects by downregulating plasmin-induced inflammatory pathways [2,3]. Moreover, the drug blocks plasmin-induced platelet activation, preserving platelets for subsequent clot formation [4]. Its efficacy and safety have been demonstrated across multiple surgical disciplines, including trauma, orthopedic, and cardiac surgery, with significant reductions in perioperative blood loss and transfusion needs [5–8]. Although potential adverse reactions such as thromboembolic events, seizures, visual disturbances, and allergic reactions have been reported, TXA’s safety profile is well established [9–12]. Contraindications to TXA use include: active thromboembolism, subarachnoid hemorrhage, known thrombophilia, recent stroke or myocardial infarction, severe renal impairment (requiring dose adjustment), and history of seizures [13].

TXA’s role in plastic surgery warrants further investigation, as even residual bleeding complications affect aesthetic outcomes and patient recovery [14–16]. A British Association of Aesthetic Plastic Surgeons anonymous online survey found that over 78% of the members surveyed used TXA regularly in their clinical practice [17]. Reduction mammaplasty was the second most common procedure, after abdominoplasty, in which surgeons used TXA, with 83% of respondents administering it. Overall, 76% administered a single IV dose of TXA on induction, 14% combined an IV dose and topical application, and the remaining 10% used either intraoperative topical administration alone, an IV dose with postoperative oral tablets, or a combination of IV, topical, and oral preparations. The most common IV TXA dose was 1 g, and the concentration of topically administered TXA ranged from 1 to 5 mg/mL. No surgeon reported increased complications associated with TXA [17]. Current evidence suggests that TXA may be effective and safe for use in reduction mammaplasty [18]. Nonetheless, the ideal route of administration, optimal dose, and appropriate duration of treatment have yet to be ascertained due to differences in study protocols and surgical techniques, which hinder the development of standardized guidelines [1,19,20]. Methodologically robust evidence from other surgical disciplines indicates that a dual-administration strategy, both systemic and local, is effective and safe for optimizing hemostatic outcomes while mitigating the risks associated with high systemic exposure [21,22]. The combined use of intravenous and locally infiltrated TXA for breast reduction has not been comprehensively studied. This study aimed to evaluate the effect of combined TXA therapy (intravenous and local infiltration) on patients undergoing modified Liacyr Ribeiro breast reduction surgery.

## Methods

A controlled, non-randomized pilot study was conducted on patients undergoing breast reduction at Department of Plastic, Reconstructive and Aesthetic Surgery of São João University Hospital between January 2018 and December 2024, all operated on by the same surgeon.

This study was approved by the Ethical Committee. All patients enrolled in this study signed informed consent documents. Eligible patients presented with breast hypertrophy and/or breast ptosis meeting criteria for breast reduction surgery. Exclusion criteria were significantly elevated operative health risks, body mass index (BMI) greater than 30 kg/m^2^, use of antiplatelet agents or anticoagulants, history of previous thromboembolism, and known allergy to tranexamic acid.

For each participant, the variables assessed included age, smoking habits, comorbidities, BMI, and weight of the surgical specimens. Primary outcomes were total and daily drain outputs. Secondary outcomes included duration of hospital stay and postoperative complications such as hemorrhagic events, tissue necrosis, wound dehiscence, and infection.

Patient data were collected prospectively from consecutive surgeries between May 2023 and December 2024 (Group B), and retrospectively from consecutive surgeries between January 2018 and May 2023, before the adoption of TXA therapy (Group A). To minimize temporal bias, several measures were implemented: (1) identical surgical technique and perioperative protocols were maintained across both periods, (2) the same surgeon performed all procedures, (3) consistent drain management protocols, (4) similar patient selection criteria were applied, and (5) the transition period was brief (May 2023) to minimize the impact of evolving surgical practice.

### Surgical Technique

**Fig. 1:**
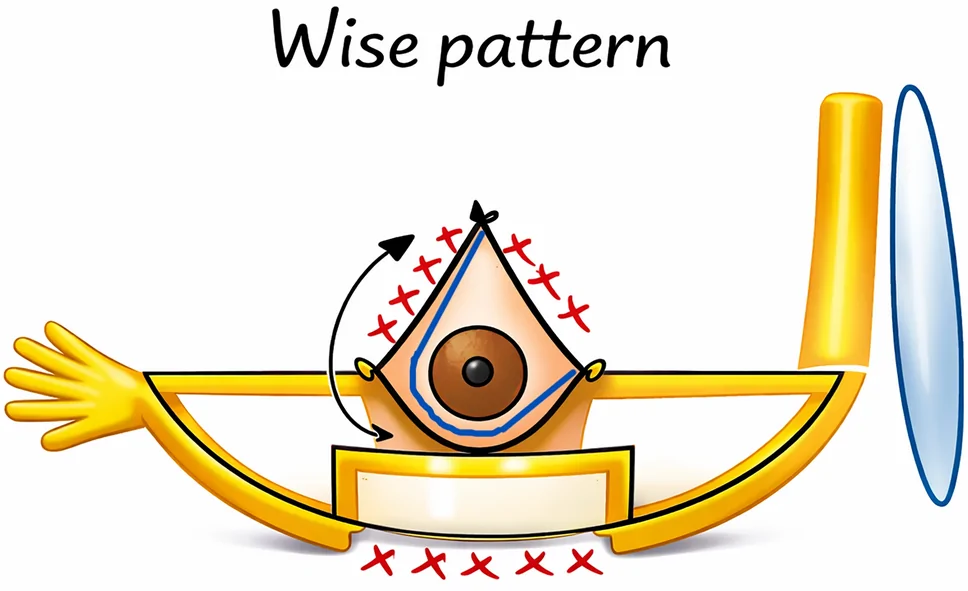
Schematic illustration of the areas of local therapy with TXA by subcutaneous injection of a 1-mg/mL vasoconstrictive solution (500 mL saline, 1 mL epinephrine 1:1000, 500 mg TXA). The areas to be infiltrated via subcutaneous injection are marked in yellow, and extend to the axilla (on the left) and over the perforators from the internal mammary artery on the right close to the sternum. The red crosses correspond to areas where the infiltration solution is not applied to preserve the vascularization of the NAC flap and the inferior lipo-glandular flap. A superomedial flap for NAC transposition is outlined with a blue line. The blue oval represents the sternum. The illustration was derived from an original hand-drawn scheme and refined using generative artificial intelligence (ChatGPT, OpenAI, GPT-5.2)

Preoperative care included enoxaparin (40 mg/d subcutaneously, starting at least 2h before surgery) and broad-spectrum antibiotics. All patients underwent breast reduction using the modified Liacyr Ribeiro technique. The surgical technique began with patient preparation and draping under general anesthesia. Equal amounts of vasoconstrictive solution were infiltrated into each breast. The standard protocol for bilateral reduction mammoplasty involves perioperative subcutaneous infiltration of an equal volume of vasoconstrictor solution in each breast, consisting of 500 mL saline and 1 mL of epinephrine (1:1000) [23]. Considering the growing body of evidence supporting the benefits of TXA in breast reduction surgery, this protocol was updated in May 2023 to incorporate combined TXA therapy. This comprises the preoperative administration of 1 g of intravenous TXA at anesthesia induction and the addition of 500 mg of TXA to the vasoconstrictor solution (concentration of 1 mg/mL TXA). Care was taken to ensure that the same amount of infiltration solution was used for both breasts (Fig. 1). From this point onward, the procedure was the same for both groups. An inferior lipo-glandular flap was designed to improve the breast shape and dissected in the central part of the lower pole of the breast in the area to be resected in the Wise pattern. The nipple–areola complex (NAC) was transposed using either a superomedial or superolateral-based flap; we preferred using the same type of pedicle on both sides. Two closed-suction drains were used in all patients, one per breast. Axillary liposuction was performed as necessary. Compression brassieres were routinely applied by the surgical team in the operating room. All patients received standard venous thromboembolism (VTE) prophylaxis according to institutional protocol. The indication was based on individual thromboembolic risk assessment and not related to TXA administration.

Drains were removed based on a volumetric criterion of less than 30 mL collected over 24 hours in each breast. A compression brassier was worn for at least 6 weeks, and strenuous activities were avoided during this period. After discharge, patients were evaluated by a senior surgeon at 7 days, 15 days, 1 month, and 6 months postoperatively.

In our practice, closed-suction drains have a reservoir with a numerical scale that cannot measure volumes of less than 5 mL, making it difficult to quantify minimal drainage output accurately. Clinical records often describe the drain output as “vestigial” indicating that it is too small to be measured precisely. To ensure consistency in data interpretation, these vestigial amounts were recorded as 0 mL. Although a small amount of fluid may be present, its volume is clinically insignificant and falls below the measurable limit of our scale.

### Statistical Analysis

Statistical analyses were performed using R statistical software (version 4.4.1, R Foundation for Statistical Computing, Vienna, Austria). Continuous variables were expressed as mean ± standard deviation or median (interquartile range). Categorical variables were presented as frequencies and percentages. Comparisons between groups for baseline characteristics were performed using the Wilcoxon rank sum test for continuous variables and Fisher’s exact test or Pearson’s Chi-squared test for categorical variables, as appropriate. Tables were created using the gtsummary package (version 2.0.3; Sjoberg et al., 2021).

An analysis of covariance (ANCOVA) was conducted to assess the relationship between group and drain output while adjusting for potential confounding variables. The ANCOVA model included group B as the primary independent variable, with age, BMI, and length of hospital stay as covariates. Estimated marginal means with 95% confidence intervals were calculated to evaluate group differences. Model assumptions were assessed using visual diagnostics, including Q-Q and residual plots for normality, homoscedasticity, and linearity.

## Results

**Fig. 2:**
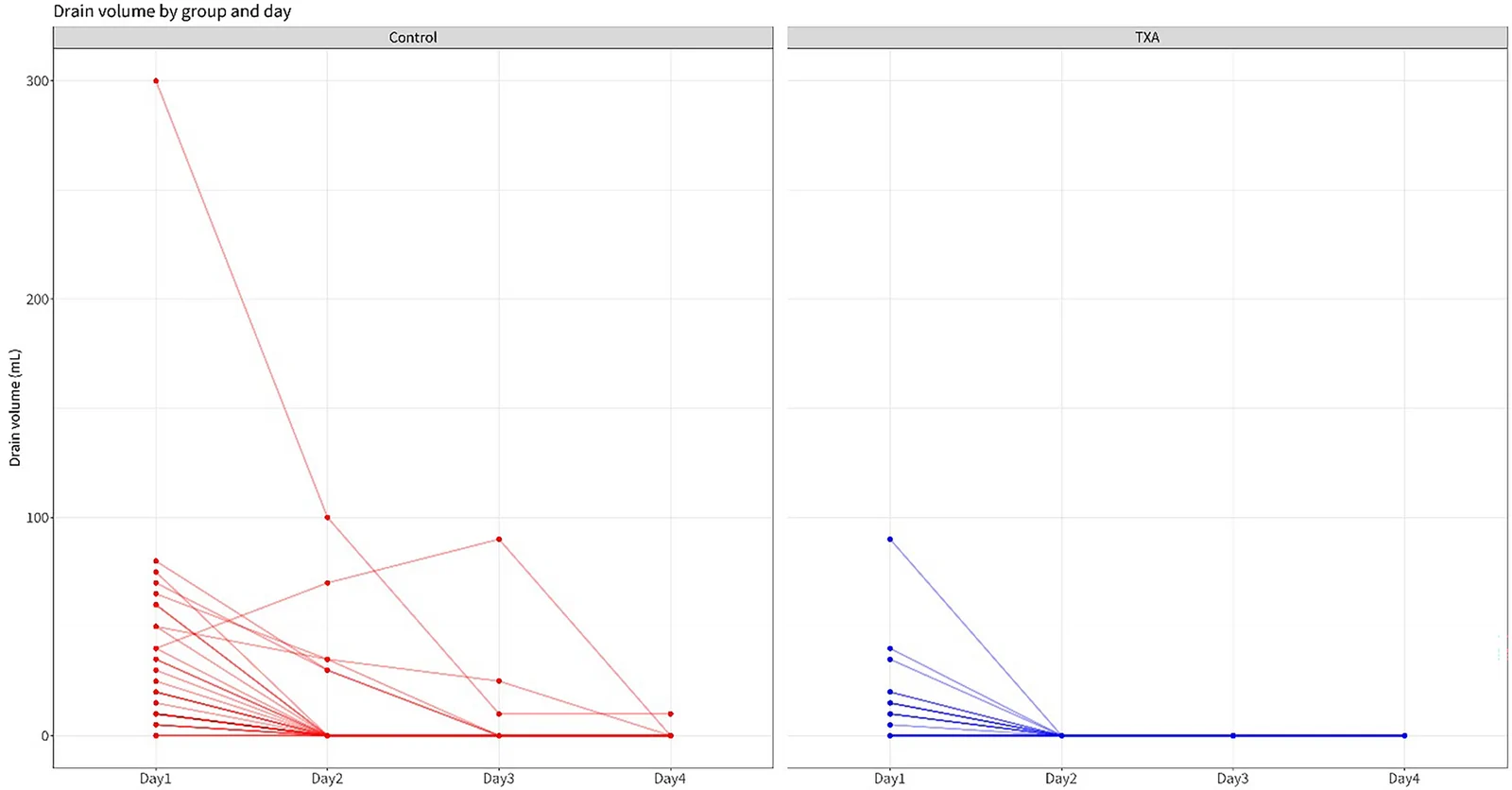
Drain output (mL) by postoperative day in the control group (red, left panel) versus the TXA group (blue, right panel). Each line represents an individual patient’s drain output trajectory over time. The y-axis represents the daily drain output, while the x-axis categorizes the measurements by day (Day 1, Day 2, Day 3, Day 4)

**Fig. 3:**
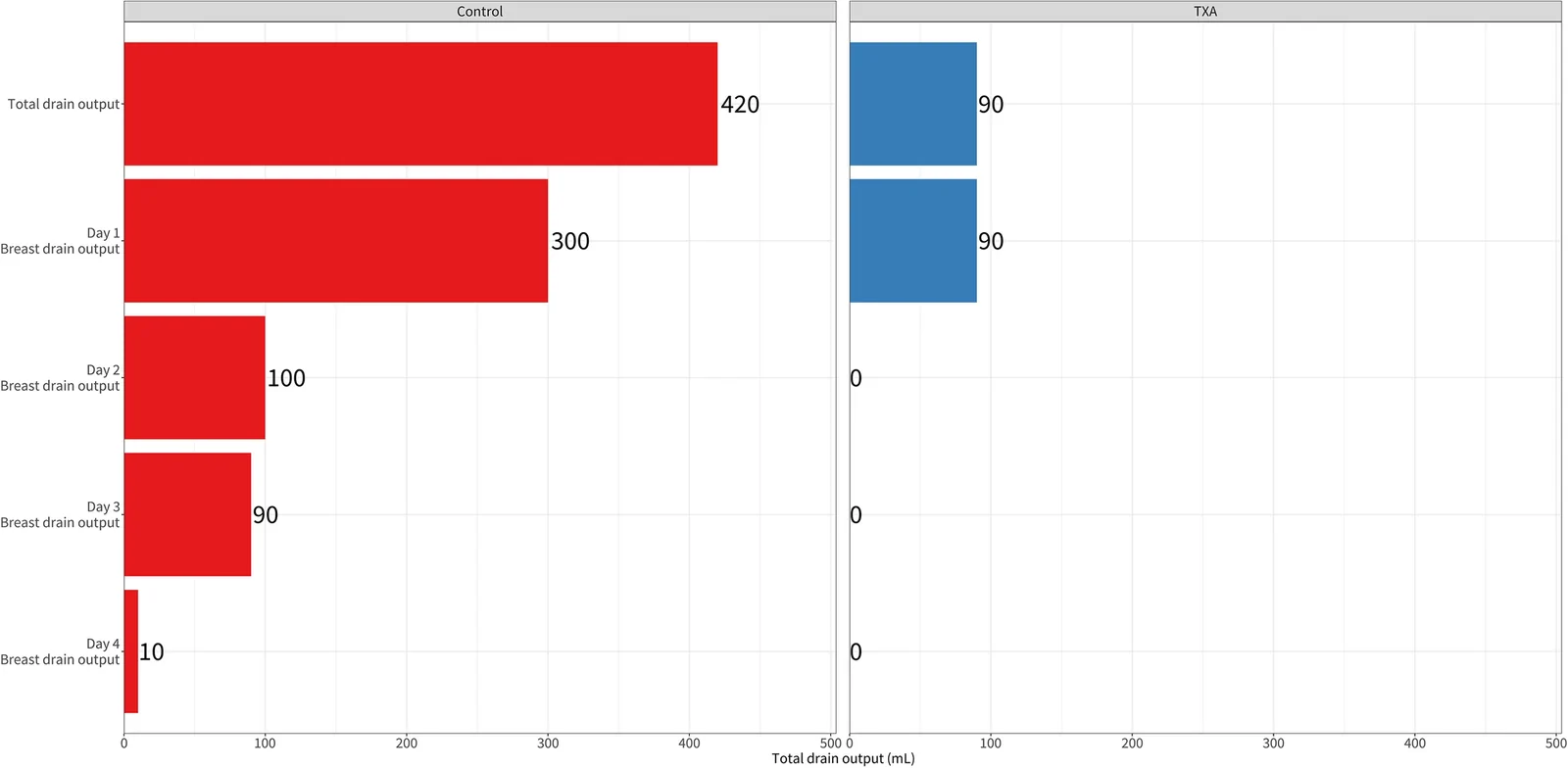
Maximum total postoperative drain output in the control and TXA groups. This bar chart compares the maximum total drain output (mL) between the control (red) and TXA (blue) groups. The x-axis represents the maximum total drain output, while the y-axis categorizes the measurements by specific time points (Day 1, Day 2, Day 3, Day 4)

Fifty-seven patients underwent bilateral modified Liacyr Ribeiro reduction mammaplasty and were included in this study: 31 in group A (no TXA) and 26 in group B (TXA). Demographic analysis revealed comparable baseline characteristics between the two groups. Both groups underwent comparable tissue resection, and the surgical indications were similarly distributed across groups (Table 1). This study yielded statistically significant findings regarding postoperative drain output (Table 2 and Fig. 2). Group B demonstrated a substantial reduction in total drain output (*p*<0.001). This reduction pattern was also observed in daily measurements. Group B consistently exhibited lower drain volumes and earlier drain removal. As illustrated in Figs. 2 and 3, the drain output in group B decreased sharply on the first postoperative day, with most patients exhibiting no measurable drainage by Day 2. In contrast, group A displayed greater variability, with several patients maintaining notable drain outputs beyond Day 2 and until Day 4. A significantly higher proportion of patients in group B had unquantifiable drainage outputs (53.8%) than in group A (9.7%) (*p*=0.002).

**Table 1:** Baseline characteristics stratified by group (*N*=57)

Characteristic	Control Group N = 31^a^	TXA Group N = 26^a^	*p*-value^b^
Age (years)	45 (38, 55)	40 (34, 52)	0.11
Active smoking	3 (9.7%)	0 (0.0%)	0.2
Comorbidities			0.11
History of diabetes mellitus	0 (0.0%)	1 (3.8%)	
History of arterial hypertension	6 (19.6%)	1 (3.8%)	
Rationale for surgery			0.4
Breast ptosis after MWL	10 (32.2%)	11 (42.3%)	
Breast hypertrophy	21 (68.8%)	15 (58.7%)	
BMI (kg/m^2^)	25.2 (23.9, 27.4)	25.2 (21.3, 26.4)	0.2
Total resected breast specimen weight (g)	490.0 ± 321.0	439.0 ± 378.0	0.4
Range	26.0 - 1330.0	50.0 - 1161.0	

^a^Median (Q1, Q3); n (%); Mean ± SD. ^b^Wilcoxon rank sum test; Fisher’s exact test; Pearson’s Chi-squared test

**Table 2:** Postoperative total and daily drain output stratified by group (*N*=57)

Outcome	Control Group N = 31^1^	TXA Group N = 26^1^	*p*-value^2^
Total drain output (mL)	52.0 ± 82.0	11.0 ± 20.0	<0.001
Min; Max	0.0; 420.0	0.0; 90.0	
Drain output after 24h on the left (mL)	25.0 ± 50.0	5.0 ± 7.0	0.001
Min; Max	0.0; 280.0	0.0; 25.0	
Drain output after 24h on the right (mL)	13.0 ± 12.0	6.0 ± 13.0	0.002
Min; Max	0.0; 40.0	0.0; 65.0	
Discharged at 24h post-op	23	23	
Drain output after 48h on the left (mL)	26.0 ± 33.0	–	0.069
Min; Max	0.0; 100.0	–	
Drain output after 48h on the right (mL)	11.0 ± 11.0	–	0.12
Min; Max	0.0; 30.0	–	
Discharged at 48h post-op	28	26	
Drain output after 72h on the left (mL)	28.0 ± 20.0	–	
Min; Max	10.0; 50.0	–	
Drain output after 72h on the right (mL)	13.0 ± 23.0	–	
Min; Max	0.0; 40.0	–	
Discharged at 72h post-op	28	26	
Drain output after 96h on the left (mL)	5.0 ± 7.0	–	
Min; Max	0.0; 10.0	–	
Drain output after 96h on the right (mL)	–	–	
Min; Max	–	–	

^1^Mean ± SD; n (Min); n (Max). ^2^Wilcoxon rank sum test; Fisher’s exact test; Pearson’s Chi-squared test

**Fig. 4:**
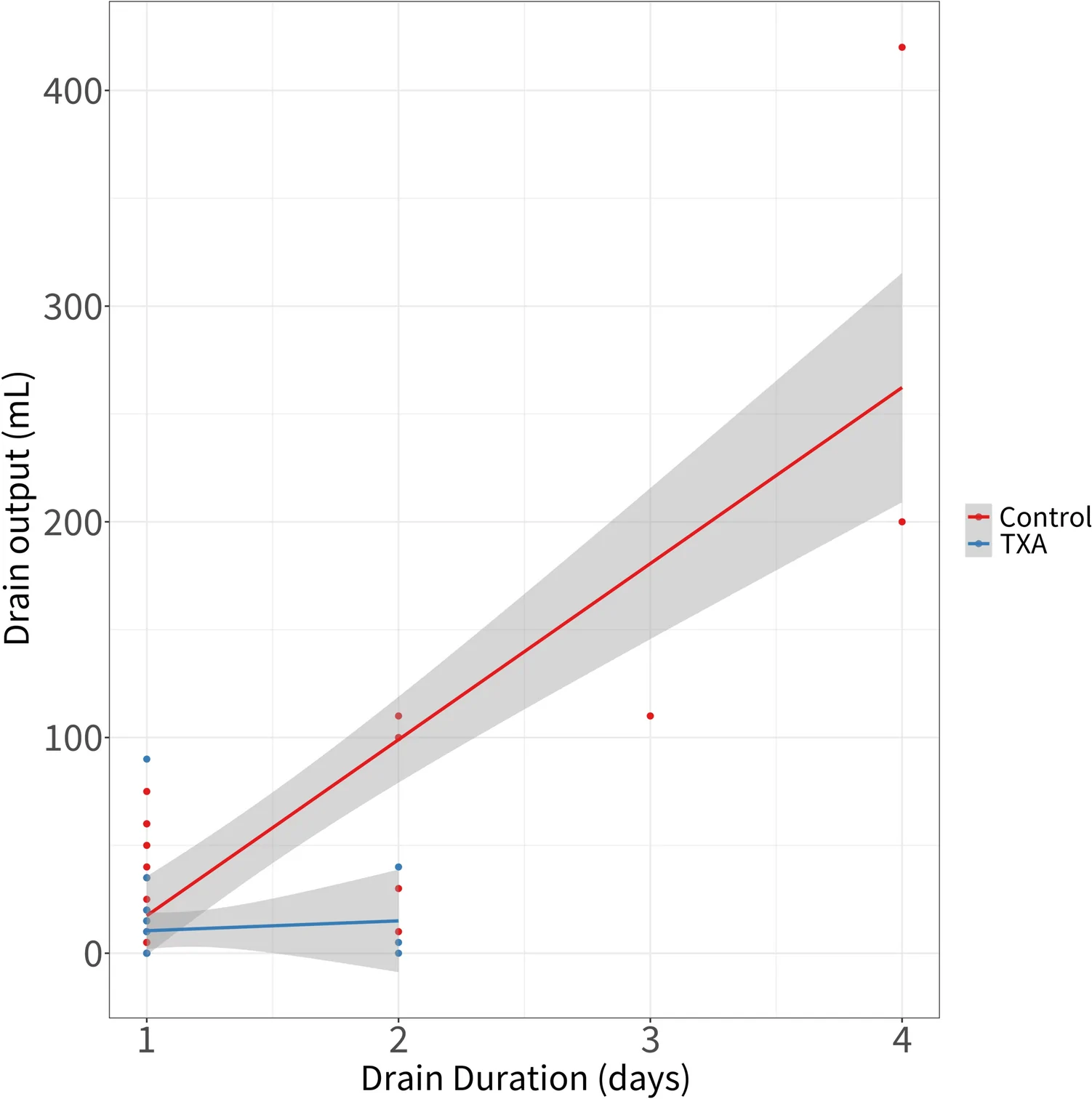
ANCOVA results assessing the effect of tranexamic acid (TXA) on drain volume over time. The x-axis represents the drain duration (days), and the y-axis represents the corresponding drain volume (mL). The red and blue lines represent the regression lines for the control and TXA groups, respectively. The gray shaded area denotes the 95% confidence interval (CI) of the fitted regression lines

The ANCOVA model (Fig. 4) revealed a statistically significant main effect of group assignment (*p*<0.001), indicating significant differences between the control and TXA groups after adjusting for covariates. Length of hospital stay also demonstrated a robust and significant effect (*p*<0.0001). BMI (*p*=0.19) was not significant. Age did not significantly contribute to outcome variability (*p*=0.20).

Estimated marginal means analysis showed adjusted mean drain outputs of 40.2 mL (95% CI [25.7, 54.8]) for controls versus 24.5 mL (95% CI [8.5, 40.6]) for TXA patients. This represents a clinically meaningful 15.7 mL reduction (39%) with non-overlapping confidence intervals confirming statistical significance (Tables S1 and S2). Most patients in both groups had a hospital stay of one day, with 74.2% in group A and 88.5% (23/26) in group B. Hospitalization exceeding two days was observed in 9.7% of group A, whereas no patients in group B required hospitalization for two or more days (Table 3).

**Table 3:** Comparison of hospital stay and drain duration between control and TXA groups

Outcome	Control Group *N* = 31^a^	TXA Group *N* = 26^a^	*p*-value^b^
Length of hospital stay (days)			0.5
1	23 (74.2%)	23 (88.5%)	
2	5 (16.1%)	3 (11.5%)	
3	1 (3.2%)	0 (0.0%)	
4	2 (6.5%)	0 (0.0%)	
Drain duration (days)			0.5
1	23 (74.2%)	23 (88.5%)	
2	5 (16.1%)	3 (11.5%)	
3	1 (3.2%)	0 (0.0%)	
4	2 (6.5%)	0 (0.0%)	
Drain duration per volumetric criterion (days)			0.025
1	23 (74.2%)	26 (100.0%)	
2	5 (16.1%)	0 (0.0%)	
3	1 (3.2%)	0 (0.0%)	
4	2 (6.5%)	0 (0.0%)	

^a^n (%). ^b^Wilcoxon rank sum test; Fisher’s exact test; Pearson’s Chi-squared test

Regarding time until drain removal (Table 3), 74.2% of patients in group A had their suction drains removed after one day, compared with 88% (23/26) in group B. Drainage for two or more days was required in 25.8% of group A, whereas only 11.5% (3/26) of group B. However, *post hoc* analysis showed that three patients in group B retained their drains beyond the anticipated timeframe despite meeting the volumetric removal criteria (<30mL/24h) because of factors unrelated to surgical complications, reflecting the clinician’s judgment. Similar cases were not observed in group A. When adjusting the time until drain removal based solely on the established volumetric criteria, all participants in group B had their drains removed on postoperative Day 1. Under these conditions, the TXA group exhibited a significantly shorter time until drain removal than the control group (*p*=0.025). In group A, 26% of patients required drainage for more than one day, highlighting a notable and statistically significant difference from group B, where no patients required drainage beyond day one.

The safety profile of TXA was favorable (Table 4). None of the groups reported major hematomas, required blood transfusions, underwent re-intervention, or had any thrombotic events during the 30-day postoperative follow-up period.

**Table 4:** Incidence of postoperative complications in control and TXA groups

Variables	Control group *N* = 31^a^	TXA group *N* = 26^a^	*p*-value^b^
Any hemorrhagic complication	1 (3.2%)	2 (7.7%)	0.6
Minor hematoma (not requiring surgical revision)	1 (3.2%)	2 (7.7%)	0.6
Major hematoma (requiring surgical revision)	0 (0.0%)	0 (0.0%)	
Need for transfusion	0 (0.0%)	0 (0.0%)	
Necrosis	0 (0.0%)	0 (0.0%)	
Infection	1 (3.2%)	1 (3.8%)	>0.9
Wound dehiscence	3 (9.7%)	3 (11.5%)	>0.9
Thromboembolic events	0 (0.0%)	0 (0.0%)	
Need for immediate re-intervention	0 (0.0%)	0 (0.0%)	
Need for unscheduled care	3 (9.7%)	3 (11.5%)	>0.9
Motive - Dehiscence	1 (33.3%)	0 (0.0%)	
Motive - Pain	1 (33.3%)	2 (66.7%)	
Motive - Wound leakage	1 (33.3%)	0 (0.0%)	

^a^n (%). ^b^Wilcoxon rank sum test; Fisher’s exact test; Pearson’s Chi-squared test

## Discussion

This study evaluated the postoperative outcomes of patients who underwent reduction mammaplasty using a modified Liacyr Ribeiro technique with a focus on the clinical benefits of combined TXA therapy (intravenous and local infiltration).

TXA significantly reduced suction drain output without increasing the complication rates. Combined TXA therapy resulted in a 77% reduction of the total drain output. Drainage resolved more rapidly in the TXA group, with most patients showing ‘vestigial’ or no output following postoperative Day 1—a pattern not seen in the control group. Notably, 53.8% of TXA-treated patients had unquantifiable drainage, enabling earlier drain removal and shorter hospital stay. All patients in the intervention group met criteria for drain removal on Day 1, whereas 25% of controls required drains beyond Day 1. These results were corroborated by ANCOVA, in which the intervention’s efficacy persisted even after controlling for covariates.

In addition to its hemostatic benefits, TXA exerts anti-inflammatory effects by inhibiting the conversion of plasminogen to plasmin and downregulating cytokine production [24,25]. This abatement of the inflammatory response to the surgical insult may explain our findings, specifically the major reduction in total and daily drain output volumes. TXA was administered only intraoperatively, as antifibrinolytic activity peaks during surgery, when fibrinolysis is highest; CRASH-2 and WOMAN trials showed early benefits without the need for postoperative extension in elective cases. Breast reduction studies confirm efficacy with intraoperative topical/IV TXA alone, without prolonged dosing [9,12,26].

No adverse events, including thromboembolism, were observed. In our cohort, TXA was not used to counteract anticoagulation nor was enoxaparin prescribed to mitigate TXA-related risk, but rather as standard VTE prophylaxis guided by individual risk stratification. Smoking is a recognized risk factor for wound healing complications and tissue necrosis in breast reduction surgery due to its vasoconstrictive and microvascular effects [27,28]. Although TXA stabilizes fibrin clots, there is no strong evidence suggesting that it increases microvascular thrombosis in otherwise healthy patients. In our cohort, smoking status was recorded and was balanced between study groups, with no increase in skin or nipple–areola complex necrosis observed in the TXA group. Multiple large-scale trials and meta-analyses, including the CRASH-2 trial (20,211 trauma patients) [9] and the ATACAS trial (4,631 cardiac surgery patients) [29], demonstrate that tranexamic acid (TXA) reduces mortality and bleeding without increasing thromboembolic events (TE) such as deep vein thrombosis, pulmonary embolism, myocardial infarction, or stroke. These findings hold across trauma and elective surgery contexts, even at standard doses (up to 100 mg/kg, later reduced to 50 mg/kg in ATACAS due to seizure concerns, primarily in open-heart procedures). In aesthetic breast surgery populations without hypercoagulable risks, TE rates with TXA remain negligible, aligning with our cohort’s zero TE events during follow-up. Operative duration was not recorded in this study, representing a potential limitation that precludes direct assessment of its influence as a confounder on thromboembolic outcomes.

Prolonged operative time is a recognized independent risk factor for perioperative thromboembolism. However, systematic reviews and meta-analyses of TXA use across surgical disciplines, including orthopedic and general procedures, demonstrate no increased thromboembolic risk with TXA administration [30].

The use of TXA in plastic surgery has increased markedly in recent years, with a growing body of research evaluating its effectiveness in preventing bleeding [31,32]. However, studies investigating the role of TXA in reducing postoperative bleeding in breast reduction surgery have yielded mixed results [1,18,20].

The retrospective study by Weissler did not find any reduction in the risk of postoperative bleeding or seroma formation requiring surgical evacuation in reduction mammaplasty patients receiving 1 g intravenous and/or topical 4% TXA (total topical TXA dosage of 3 g) via spray applicator and retrogradely through the drain [33]. This study used three different routes of TXA administration: intravenous, topical, and intravenous plus topical. Additionally, two different surgical techniques were used: superomedial and inferior pedicles; however, their distribution between the groups was uneven (*p*<0.0001). The placement of closed-suction drains also varied between groups, depending on the surgeon’s preference.

Nichols reported no decrease in postoperative hematoma and seroma, intraoperative blood loss, or operative time with local infiltration of TXA (3 mg/mL), corresponding to an estimated total TXA dose between 600 and 900 mg [34]. The intervention and the control groups received a solution with 1 L of lactated Ringer’s solution, 300 mg of lidocaine, and 1 mg of epinephrine. In contrast, the intervention group received 1500 mg TXA added to the tumescent solution. Each patient received approximately 200–300 ml of tumescent solution infiltrated into each breast. Nevertheless, this study reported beneficial effects of TXA on suture granuloma (*p*=0.05), wound dehiscence (*p*=0.016), and drain duration (*p*=0.033).

Recent studies reported other evidence for the use of TXA. Sipos found that rinsing each breast with 500 mg TXA—25 ml of a 20 mg/ml TXA solution—just before wound closure reduced the incidence of postoperative hematoma to one-tenth of that observed in the control group [35]. Another study by Om showed that 1 g intravenous TXA at the beginning of the operation significantly decreased the incidence of both major and minor postoperative hematoma (*p*=0.0143) [36]. Neither Sipos [35] nor Om [36] used epinephrine infiltration.

Overall, current evidence from a recently published systematic review suggests that TXA may be effective and safe for use in the reduction of mammaplasty. TXA in breast reduction is not risk-free but has a consistently favorable profile. Meta-analyses of reduction mammaplasty show no increased in TE events, seizures, or further complications with TXA therapy [18,37].

Our study aligns with the existing methodologically robust evidence on the impact of TXA on postoperative drain output in breast reduction surgery. Ausen conducted a double-blinded randomized controlled trial (RCT) and reported a 39 % reduction in drain volume with topical moistening of 500 mg TXA before wound closure (*p*=0.038) [38]. Recently, Magni conducted a retrospective study involving 157 patients who underwent reduction mammaplasty using a systemic TXA protocol consisting of 1 g intravenous TXA every 8 h for the first 24 h, followed by 1 g oral TXA every 8 h for the subsequent 24 h. This protocol resulted in a 22% reduction of total drain output (*p*=0.012), enabling significantly earlier drain removal by 0.6 days (*p*=0.0001) [39]. Isolated local infiltration of TXA into the breast tissue was not advantageous, as demonstrated by Nichols using a 3-mg/mL TXA solution. However, when TXA is used in combined therapy, specifically systemic intravenous TXA at induction associated with breast infiltration with a TXA solution, different outcomes have been observed. Abboud performed an RCT in which 500 mg intravenous TXA during induction was associated with local infiltration of a solution containing 500 mg TXA per L of normal saline. The control group received breast infiltration with normal saline only. This study demonstrated that the combined use of locally infiltrated and IV TXA reduced blood loss during surgery (*p*=0.0002) and postoperative ecchymosis, as evaluated by photographic analysis [40].

Considering the studies comparing different TXA therapies and their specific effects on drain output, our combined protocol achieved an estimated 79% reduction. This surpasses the 39% reduction reported with topical TXA in Ausen’s study [38] and the 22% reduction observed in Magni’s 48 h systemic protocol [39]. The results of our investigation further validate combined TXA therapy as advantageous for other outcomes, including reduced daily drain output, shorter time to drain removal, and decreased duration of hospital stay.

The use of drains in breast reduction surgery remains a subject of debate. An online survey of members of the International Society of Plastic Regenerative and Aesthetic Surgery revealed that, depending on resection weight, between 43 and 57% of surgeons consistently use drains for breast reduction procedures [41]. Nevertheless, the clinical practice guidelines from the American Society of Plastic Surgeons do not recommend the routine use of closed-suction drains in breast reduction. This recommendation was recently challenged by Behroozian [35], who highlighted the methodological limitations of RCTs on which the guidelines are based [36–38]. Indeed, closed-suction drains do not significantly reduce postoperative hematoma or seroma and even significantly increase the risk of postoperative surgical site infection (*p*=0.011) [42].

Our findings indicate that combined TXA therapy effectively reduced postoperative drain fluid production, with all treated patients meeting removal criteria by Day 1, suggesting the potential to eliminate the need for drains. This observation is even more relevant considering that 53.8% of the patients presented unquantifiable volumes in their suction drain reservoirs. Consistent volume reduction, absence of adverse events, and shorter hospital stays support the integration of combined TXA therapy into the surgical protocol for the reduction of mammaplasty.

This study should be interpreted in light of its design as a controlled, non-randomized pilot study. Although all procedures were performed by the same surgeon using a standardized technique, the absence of randomization and the relatively small sample size (57 patients) limit the level of evidence and preclude definitive causal inferences. The magnitude of effect observed in this pilot cohort supports the feasibility and potential clinical relevance of combined TXA therapy in reduction mammaplasty; however, methodological validation through adequately sized RCTs is needed to validate TXA’s effects on bleeding, drain use, and long-term safety, including drain-free protocols. Our single-surgeon design, while ensuring technical consistency, limits the generalizability of the findings to other surgeons and institutions. The small sample size of 57 patients also affected our statistical power, particularly for detecting differences in rare complications. As a pilot study, this limitation was anticipated, and our primary goal was to evaluate the feasibility of combined TXA therapy and provide preliminary evidence to support future investigation.

Finally, our control group had already received epinephrine-containing infiltration, which may have diminished the apparent benefit of TXA. Epinephrine-containing solutions have been shown to significantly reduce intraoperative blood loss without affecting operative time or postoperative drainage [23,43], explaining the relatively low complication rates in both groups and underscoring the need for larger studies to detect meaningful differences in safety outcomes.

Despite these limitations, our results establish a foundation for future randomized controlled trials with larger sample sizes by offering valuable preliminary evidence supporting the efficacy of combined TXA therapy in breast reduction surgery.

## Conclusion

The administration of combined TXA therapy resulted in a significant and clinically meaningful reduction in the postoperative drain output. This reduction facilitates earlier drain removal and may ultimately contribute to the broader adoption of drain-free breast reduction surgery.

## Supplementary Information

Below is the link to the electronic supplementary material.

Supplementary file1 (DOCX 17 kb)

## References

[1] Calpin GG, McAnena PF, Davey MG, Calpin P, Kerin MJ, McInerney N, et al. The role of tranexamic acid in reducing post-operative bleeding and seroma formation in breast surgery: a meta-analysis. Surgeon. 2023;21:e183–94. 10.1016/j.surge.2022.11.005 36572609

[2] CRASH-2 Collaborators, Shakur H, Roberts I, Bautista R, Caballero J, Coats T, Dewan Y, El-Sayed H, Gogichaishvili T, Gupta S, Herrera J, Hunt B, Iribhogbe P, Izurieta M, Khamis H, Komolafe E, Marrero MA, Mejia-Mantilla J, Miranda J, Morales C, Olaomi O, Olldashi F, Perel P, Peto R, Ramana PV, Ravi RR, Yutthakasemsunt S. Effects of tranexamic acid on death, vascular occlusive events, and blood transfusion in trauma patients with significant haemorrhage (CRASH-2): a randomised, placebo-controlled trial. Lancet 2010;376: 23-32 10.1016/S0140-6736(10)60835-5 20554319

[3] Poeran J, Rasul R, Suzuki S, Danninger T, Mazumdar M, Opperer M, et al. Tranexamic acid use and postoperative outcomes in patients undergoing total hip or knee arthroplasty in the United States: retrospective analysis of effectiveness and safety. BMJ. 2014;349:g4829. 10.1136/bmj.g4829 PMC4130961 25116268

[4] Hunt BJ. The current place of tranexamic acid in the management of bleeding. Anaesthesia. 2015;70:50-e18. 10.1111/anae.12910 25440395

[5] Lier H, Maegele M, Shander A. Tranexamic acid for acute hemorrhage: a narrative review of landmark studies and a critical reappraisal of its use over the last decade. Anesth Analg. 2019;129:1574–84. 10.1213/ANE.0000000000004389 31743178

[6] Zhang Y, Bai Y, Chen M, Zhou Y, Yu X, Zhou H, et al. The safety and efficiency of intravenous administration of tranexamic acid in coronary artery bypass grafting (CABG): a meta-analysis of 28 randomized controlled trials. BMC Anesthesiol. 2019;19:104. 10.1186/s12871-019-0761-3 PMC6567423 31195987

[7] Xiong H, Liu Y, Zeng Y, Wu Y, Shen B. The efficacy and safety of combined administration of intravenous and topical tranexamic acid in primary total knee arthroplasty: a meta-analysis of randomized controlled trials. BMC Musculoskelet Disord. 2018;19:321. 10.1186/s12891-018-2181-9 PMC6129000 30193586

[8] Hong P, Liu R, Rai S, Liu J, Ding Y, Li J. Does tranexamic acid reduce the blood loss in various surgeries? An umbrella review of state-of-the-art meta-analysis. Front Pharmacol. 2022;13:887386. 10.3389/fphar.2022.887386 PMC9160460 35662737

[9] Roberts I, Shakur H, Coats T, Hunt B, Balogun E, Barnetson L, et al. The CRASH-2 trial: a randomised controlled trial and economic evaluation of the effects of tranexamic acid on death, vascular occlusive events and transfusion requirement in bleeding trauma patients. Health Technol Assess. 2013;17:1–79. 10.3310/hta17100 PMC4780956 23477634

[10] Myles PS, Smith JA, Forbes A, Silbert B, Jayarajah M, Painter T, et al. Tranexamic acid in patients undergoing coronary-artery surgery. N Engl J Med. 2017;376:136–48. 10.1056/NEJMoa1606424 27774838

[11] Ker K, Beecher D, Roberts I. Topical application of tranexamic acid for the reduction of bleeding. Cochrane Database Syst Rev. 2013. 10.1002/14651858.cd010562. PMC12053458 23881695

[12] Ker K, Roberts I. Tranexamic acid for surgical bleeding. BMJ. 2014;349:g4934. 10.1136/bmj.g4934 25122635

[13] Habarth-Morales TE, Isch E, Zavitsanos AP, Wride WM, Davis HD, Rios-Diaz AJ, et al. The use of tranexamic acid in breast reduction and abdominoplasty: a review of a multicenter federated electronic health record database. Aesthet Surg J Open Forum. 2024;6:ojae077. 10.1093/asjof/ojae077 PMC11487907 39430211

[14] Kaoutzanis C, Winocour J, Gupta V, Ganesh Kumar N, Sarosiek K, Wormer B, et al. Incidence and risk factors for major hematomas in aesthetic surgery: analysis of 129,007 patients. Aesthet Surg J. 2017;37:1175–85. 10.1093/asj/sjx062 28398469

[15] Hood K, Ganesh Kumar N, Kaoutzanis C, Higdon KK. Hematomas in aesthetic surgery. Aesthet Surg J. 2018;38:1013–25. 10.1093/asj/sjx236 29474524

[16] Ching S, Thoma A, McCabe RE, Antony MM. Measuring outcomes in aesthetic surgery: a comprehensive review of the literature. Plast Reconstr Surg. 2003;111:469–80. 10.1097/01.PRS.0000036041.67101.48 12496620

[17] Wokes JET, Erdmann MWH, McLean NR. The role of tranexamic acid in aesthetic plastic surgery: a survey of the British association of aesthetic plastic surgeons. Aesthet Surg J. 2021;41:244–9. 10.1093/asj/sjaa149 32505129

[18] Pontes A, Barreiro D, Costa-Ferreira A. The impact of tranexamic acid administration in reduction mammaplasty: a systematic review. Ann Plast Surg. 2025;94:370–7. 10.1097/SAP.0000000000004184 39787391

[19] Hashemi ASA, Hussein SM, Alshehab ZH, Al Qurashi AA, Kreutz-Rodrigues L, Sharaf BA. Is topical tranexamic acid effective in reducing hematoma and seroma in breast surgery? A systematic review and meta-analysis. Plast Reconstr Surg Glob Open. 2025;13:e6442. 10.1097/GOX.0000000000006442 PMC11737497 39823029

[20] Liechti R, van de Wall BJM, Hug U, Fritsche E, Franchi A. Tranexamic acid use in breast surgery: a systematic review and meta-analysis. Plast Reconstr Surg. 2023;151:949–57. 10.1097/PRS.0000000000010071 36729428

[21] Shang J, Wang H, Zheng B, Rui M, Wang Y. Combined intravenous and topical tranexamic acid versus intravenous use alone in primary total knee and hip arthroplasty: a meta-analysis of randomized controlled trials. Int J Surg. 2016;36:324–9. 10.1016/j.ijsu.2016.11.033 27856355

[22] Cao Z, Li Q, Guo J, Li Y, Wu J. Optimal administration strategies of tranexamic acid to minimize blood loss during spinal surgery: results of a Bayesian network meta-analysis. Ann Med. 2022;54:2053–63. 10.1080/07853890.2022.2101687 PMC9307111 35862264

[23] Hardwicke JT, Jordan RW, Skillman JM. Infiltration of epinephrine in reduction mammaplasty: a systematic review of the literature. Plast Reconstr Surg. 2012;130:773–8. 10.1097/PRS.0b013e318262f085 23018690

[24] Jimenez JJ, Iribarren JL, Lorente L, Rodriguez JM, Hernandez D, Nassar I, et al. Tranexamic acid attenuates inflammatory response in cardiopulmonary bypass surgery through blockade of fibrinolysis: a case control study followed by a randomized double-blind controlled trial. Crit Care. 2007;11:R117. 10.1186/cc6173 PMC2246206 17988379

[25] Ng W, Jerath A, Wąsowicz M. Tranexamic acid: a clinical review. Anaesthesiol Intensive Ther. 2015;47:339–50. 10.5603/AIT.a2015.0011 25797505

[26] WOMAN-2 Trial Collaborators. The effect of tranexamic acid on postpartum bleeding in women with moderate and severe anaemia (WOMAN-2): an international, randomised, double-blind, placebo-controlled trial. The Lancet, 2024;404(10463):1645-165610.1016/S0140-6736(24)01749-539461792

[27] Hillam JS, Borsting EA, Chim JH, Thaller SR. Smoking as a risk factor for breast reduction: an analysis of 13,503 cases. J Plast Reconstr Aesthet Surg. 2017;70:734–40. 10.1016/j.bjps.2016.12.012 28237520

[28] Chan LK, Withey S, Butler PE. Smoking and wound healing problems in reduction mammaplasty: is the introduction of urine nicotine testing justified? Ann Plast Surg. 2006;56:111–5. 10.1097/01.sap.0000197635.26473.a2 16432315

[29] Myles PS, Smith JA, Kasza J, Silbert B, Jayarajah M, Painter T, et al. Tranexamic acid in coronary artery surgery: one-year results of the aspirin and tranexamic acid for coronary artery surgery (ATACAS) trial. J Thorac Cardiovasc Surg. 2019;157:644-652.e649. 10.1016/j.jtcvs.2018.09.113 30459103

[30] Taeuber I, Weibel S, Herrmann E, Neef V, Schlesinger T, Kranke P, et al. Association of intravenous tranexamic acid with thromboembolic events and mortality: a systematic review, meta-analysis, and meta-regression. JAMA Surg. 2021;156:e210884. 10.1001/jamasurg.2021.0884 PMC8047805 33851983

[31] Laikhter E, Comer CD, Shiah E, Manstein SM, Bain PA, Lin SJ. A systematic review and meta-analysis evaluating the impact of tranexamic acid administration in aesthetic plastic surgery. Aesthet Surg J. 2022;42:548–58. 10.1093/asj/sjab333 34486647

[32] Elena Scarafoni E. A systematic review of tranexamic acid in plastic surgery: What’s new? Plast Reconstr Surg Glob Open. 2021;9:e3172. 10.1097/GOX.0000000000003172 PMC8062149 33907653

[33] Weissler JM, Kuruoglu D, Antezana L, Curiel D, Kerivan L, Alsayed A, et al. Efficacy of tranexamic acid in reducing seroma and hematoma formation following reduction mammaplasty. Aesthet Surg J. 2022;42:616–25. 10.1093/asj/sjab399 35029651

[34] Nichols G, Andrade E, Gregoski M, Herrera FA, Armstrong MB. Local infiltration of tranexamic acid in breast reduction for symptomatic macromastia. Ann Plast Surg. 2023;90:S366–70. 10.1097/SAP.0000000000003456 PMC11166195 36856723

[35] Sipos K, Kämäräinen S, Kauhanen S. Topical tranexamic acid reduces postoperative hematomas in reduction mammaplasties. J Plast Reconstr Aesthet Surg. 2023;83:172–9. 10.1016/j.bjps.2023.04.039 37276736

[36] Om A, Marxen T, Kebede S, Losken A. The usage of intravenous tranexamic acid in reduction mammaplasty safely reduces hematoma rates. Ann Plast Surg. 2023;90:S371–4. 10.1097/SAP.0000000000003296 PMC10578999 36729851

[37] Fung E, Montalmant KE, Roth JM, Godek M, Li JH, Yu BZ, et al. Utility of tranexamic acid in reduction mammaplasty: a systematic review and meta-analysis. Aesthetic Plast Surg. 2025;49:6055–65. 10.1007/s00266-025-05052-y 40571751

[38] Ausen K, Fossmark R, Spigset O, Pleym H. Randomized clinical trial of topical tranexamic acid after reduction mammoplasty. Br J Surg. 2015;102:1348–53. 10.1002/bjs.9878 PMC4600231 26349843

[39] Magni S, Guggenheim L, Fournier G, Parodi C, Pagnamenta A, Schmauss D, et al. The effects of systemic tranexamic acid administration on drainage volume, length of hospital stay, and postoperative complications in reduction mammaplasty. J Clin Med. 2024. 10.3390/jcm14010151. PMC11720834 39797232

[40] Abboud NM, Kapila AK, Abboud S, Yaacoub E, Abboud MH. The Combined effect of intravenous and topical tranexamic acid in liposuction: a randomized double-blinded controlled trial. Aesthet Surg J Open Forum. 2021;3:ojab002. 10.1093/asjof/ojab002 PMC8240743 34212138

[41] Broer PN, Forte AJ, Topka C, Richter DF, Colombo M, Aung T, et al. Current trends in breast reduction: an international analysis. Eur J Plast Surg. 2022;45:421–8.

[42] Kalmar CL, White-Dzuro CG, Mok JW, Perdikis G. Reduction mammaplasty: closed suction drains do not reduce hematoma or seroma but increase infection risk. Ann Plast Surg. 2025;94:152–6. 10.1097/SAP.0000000000004153 39841897

[43] Soueid A, Nawinne M, Khan H. Randomized clinical trial on the effects of the use of diluted adrenaline solution in reduction mammaplasty: same patient, same technique, same surgeon. Plast Reconstr Surg. 2008;121:30e–3e. 10.1097/01.prs.0000299298.26376.9f 18317083

