# Supplementary material for: Pilot Study of Combined Tranexamic Acid Therapy in Breast Reduction – Intravenous and Local Infiltration
Source: Aesthetic Plast Surg. 2026 Aug 21;50(19):7935–44. doi: 10.1007/s00266-026-06024-6 (PMC13710680; doi:10.1007/s00266-026-06024-6)
Supplement: Supplementary file 1 [file 266_2026_6024_MOESM1_ESM.docx]

**Supplementary Material**

**Analysis of Covariance (ANCOVA)**

The analysis of covariance was performed to assess the effect of tranexamic acid on total drain volume while controlling for potential confounding variables. The results are presented below.

*Table S1. Analysis of Covariance for Total Drain Volume*

| Source | df | Sum Sq | Mean Sq | F value | P-value |
| --- | --- | --- | --- | --- | --- |
| Group | 1 | 18.256 | 18.256 | 10.928 | 0.00172 |
| Age | 1 | 1.656 | 1.656 | 0.991 | 0.32405 |
| BMI | 1 | 3.616 | 3.616 | 2.164 | 0.14726 |
| Procedure Duration | 1 | 82.109 | 82.109 | 49.148 | < 0.001 |
| Residuals | 52 | 86.874 | 1.671 | - | - |

*Table S2. Estimated Marginal Means by Treatment Group*

| Group | Estimate (mL) | Standard Error | df | 95% CI Lower | 95% CI Upper |
| --- | --- | --- | --- | --- | --- |
| Control | 38.3 | 7.60 | 52 | 23.01 | 53.5 |
| Intervention | 22.3 | 8.35 | 52 | 5.51 | 39.0 |

Note: Results are adjusted for covariates (age, BMI, and procedure duration)

Analysis of Variance Components:

- The model explained 54.8% of the total variance in drain volume

- Procedure duration was the strongest predictor (F = 49.148, P < 0.001)

- Treatment group showed significant effect (F = 10.928, P = 0.00172)

Effect Sizes:

- Group effect: η²p = 0.174 (large effect)

- Procedure duration: η²p = 0.486 (large effect)

- Age: η²p = 0.019 (small effect)

- BMI: η²p = 0.040 (small effect)
